# P-2135. Assessing Diagnostic Capabilities and Treatment Accessibility for Invasive Fungal Infections in the Balkan Region

**DOI:** 10.1093/ofid/ofae631.2290

**Published:** 2025-01-29

**Authors:** Jon Salmanton-Garcia, Aleksandra Barać, Oliver A Cornely, Nikola Pantić

**Affiliations:** University Hospital Cologne, Cologne, Germany, Cologne, Nordrhein-Westfalen, Germany; University Clinical Center of Serbia, Belgrade, Vojvodina, Serbia; University of Cologne, Faculty of Medicine and University Hospital Cologne, Cologne, Nordrhein-Westfalen, Germany; x, Belgrade, Vojvodina, Serbia

## Abstract

**Background:**

Ongoing progress in diagnosing and treating invasive fungal infections (IFI) faces challenges due to economic inequalities. Moreover, IFI prevalence and features vary between regions and countries. This study aims to assess the current diagnostic and treatment resources for IFIs in the Balkan region.

**Methods:**

Data from Albania, Bosnia and Herzegovina, Bulgaria, Croatia, Greece, Kosovo, Moldova, Montenegro, North Macedonia, Serbia, Slovenia, and Romania were collected. The survey investigated participating institutions' traits, their views on IFIs, and the accessibility of diagnostic tools like microscopy, culture, serology, antigen detection, molecular testing, and therapeutic drug monitoring.

**Results:**

Results from the survey revealed that among the 47 representatives from medical centres in the Balkans, 38% considered the incidence of IFIs to be low, while 34% perceived it as very low. Candida spp. was reported as the causative agent of IFIs in all surveyed centres (100%), followed by Aspergillus spp. (72%) and Cryptococcus spp. (43%). Diagnostic resources in the centres were as follows: 98% had access to culture and microscopy, and 93% had susceptibility testing technology. Antigen detection tests were used in 81% of the centres, while PCR tests were available in fewer centres: Aspergillus PCR (48%), Candida PCR (34%), Pneumocystis PCR (43%), and Mucorales PCR (19%). Among antifungal treatments, triazoles were the most prevalent, with 96% of centres offering them, followed by echinocandins (79%), mainly micafungin (77%), and amphotericin B (68%), primarily in its liposomal form (57%). Therapeutic drug monitoring was reported in 30% of surveyed centres, including voriconazole in all centres (100%), posaconazole in 71% of centres, itraconazole in 64% of centres, and flucytosine in 43% of centres.

**Conclusion:**

In conclusion, mycology laboratories in Balkan centres are generally well-equipped, but molecular diagnostic tools are less accessible, present in fewer than half of the surveyed centres. Triazoles are the most accessible antifungal class, while other options like echinocandins, amphotericin B, flucytosine, and terbinafine are less widely available. The survey remains open to gather more insights from additional sites in the region.

**Disclosures:**

Oliver A. Cornely, Prof. Dr., Abbott: Honoraria|Abbvie: Advisor/Consultant|Abbvie: Honoraria|AiCuris: Advisor/Consultant|Akademie fur Infektionmedizin: Honoraria|Al-Jazeera Pharmaceuticals/Hikma: Honoraria|amedes: Honoraria|AstraZeneca: Honoraria|Basilea: Advisor/Consultant|Biocon: Advisor/Consultant|BMBF: Grant/Research Support|Boston Strategic Partners: Advisor/Consultant|CIdara: Advisor/Consultant|CIdara: Expert Testimony|CIdara: Grant/Research Support|CIdara: Participation on a DRC or DSMB|CoRe Consulting: Stocks/Bonds (Private Company)|Deutscher Arzteverlag: Honoraria|DZIF: Grant/Research Support|EasyRadiology: Stocks/Bonds (Private Company)|EU-DG RTD: Grant/Research Support|F2G: Grant/Research Support|Gilead: Advisor/Consultant|Gilead: Grant/Research Support|Gilead: Honoraria|Grupo Biotoscana/United Medical/Knight: Honoraria|GSK: Advisor/Consultant|GSK: Honoraria|IQVIA: Advisor/Consultant|IQVIA: Participation on a DRC or DSMB|Janssen: Advisor/Consultant|Janssen: Participation on a DRC or DSMB|Matinas: Advisor/Consultant|MedPace: Advisor/Consultant|MedPace: Grant/Research Support|MedPace: Participation on a DRC or DSMB|Medscape/WebMD: Honoraria|MedUpdate: Honoraria|Menarini: Advisor/Consultant|Moderna: Honoraria|Molecular Partners: Advisor/Consultant|MSD: Grant/Research Support|MSD: Honoraria|MSG-ERC: Advisor/Consultant|Mundipharma: Advisor/Consultant|Mundipharma: Grant/Research Support|Mundipharma: Honoraria|Noscendo: Honoraria|Noxxon: Advisor/Consultant|Octapharma: Advisor/Consultant|Octapharma: Grant/Research Support|Pardes: Advisor/Consultant|Partner Therapeutics: Advisor/Consultant|Patent: US18/562644|Paul-Martini-Stiftung: Honoraria|Pfizer: Advisor/Consultant|Pfizer: Grant/Research Support|Pfizer: Honoraria|PSI: Advisor/Consultant|PSI: Participation on a DRC or DSMB|Pulmocide: Participation on a DRC or DSMB|Sandoz: Honoraria|Scynexis: Advisor/Consultant|Scynexis: Grant/Research Support|Seqirus: Advisor/Consultant|Seqirus: Honoraria|Seres: Advisor/Consultant|Shionogi: Advisor/Consultant|Shionogi: Honoraria|streamedup!: Honoraria|The Prime Meridian Group: Advisor/Consultant|Touch Independent: Honoraria|Vitis: Honoraria

